# The Potential of Dutasteride for Treating Multidrug-Resistant *Candida auris* Infection

**DOI:** 10.3390/pharmaceutics16060810

**Published:** 2024-06-14

**Authors:** J. Francis Borgio, Noor B. Almandil, Prathas Selvaraj, J. Sherlin John, Rahaf Alquwaie, Eman AlHasani, Norah F. Alhur, Razan Aldahhan, Reem AlJindan, Dana Almohazey, Sarah Almofty, T. Stalin Dhas, Sayed AbdulAzeez

**Affiliations:** 1 Department of Genetic Research, Institute for Research and Medical Consultation (IRMC), Imam Abdulrahman Bin Faisal University, Dammam 31441, Saudi Arabia; fbalexander@iau.edu.sa (J.F.B.); norah.f.s.2@gmail.com (N.F.A.); raldahhan@iau.edu.sa (R.A.); 2 Department of Clinical Pharmacy Research, Institute for Research and Medical Consultation (IRMC), Imam Abdulrahman Bin Faisal University, Dammam 31441, Saudi Arabia; 3 Entomology Research Unit (ERU), Department of Zoology, St. Xavier’s College (Autonomous), Palayamkottai, Affiliated to Manonmaniam Sundaranar University, Tirunelveli 627002, Tamil Nadu, India; drselvabernad@gmail.com (P.S.); zoojohnsxc@gmail.com (J.S.J.); 4 Master Program of Biotechnology, Institute for Research and Medical Consultations (IRMC), Imam Abdulrahman Bin Faisal University, Dammam 31441, Saudi Arabia or 2230500195@iau.edu.sa (R.A.); or 2220500126@iau.edu.sa (E.A.); 5 Department of Microbiology, College of Medicine, Imam Abdulrahman Bin Faisal University, Dammam 40017, Saudi Arabia; raljindan@iau.edu.sa; 6 Department of Stem Cell Research, Institute for Research and Medical Consultations (IRMC), Imam Abdulrahman Bin Faisal University, Dammam 31441, Saudi Arabia; daaalmohazey@iau.edu.sa (D.A.); saalmofty@iau.edu.sa (S.A.); 7 Centre for Ocean Research (DST—FIST Sponsored Centre), MoES-Earth Science & Technology Cell, Sathyabama Institute of Science and Technology, Chennai 600119, India; stalindhas.cor@sathyabama.ac.in

**Keywords:** virtual screening, molecular dynamics simulations, dutasteride, *Candida auris*, drug repurposing, biofilm, planktonic growth, antifungal drugs, metabolites

## Abstract

Novel antifungal drugs are urgently needed to treat candidiasis caused by the emerging fungal multidrug-resistant pathogen *Candida auris*. In this study, the most cost-effective drug repurposing technology was adopted to identify an appropriate option among the 1615 clinically approved drugs with anti-*C. auris* activity. High-throughput virtual screening of 1,3-beta-glucanosyltransferase inhibitors was conducted, followed by an analysis of the stability of 1,3-beta-glucanosyltransferase drug complexes and 1,3-beta-glucanosyltransferase–dutasteride metabolite interactions and the confirmation of their activity in biofilm formation and planktonic growth. The analysis identified dutasteride, a drug with no prior antifungal indications, as a potential medication for anti-*auris* activity in seven clinical *C. auris* isolates from Saudi Arabian patients. Dutasteride was effective at inhibiting biofilm formation by *C. auris* while also causing a significant reduction in planktonic growth. Dutasteride treatment resulted in disruption of the cell membrane, the lysis of cells, and crushed surfaces on *C. auris*, and significant (*p*-value = 0.0057) shrinkage in the length of *C. auris* was noted at 100,000×. In conclusion, the use of repurposed dutasteride with anti-*C*. *auris* potential can enable rapid recovery in patients with difficult-to-treat candidiasis caused by *C. auris* and reduce the transmission of nosocomial infection.

## 1. Introduction 

After its discovery in Japan in 2009, *Candida auris*, a multidrug-resistant (MDR) fungus, was the first fungal human pathogen to be flagged globally as an imminent and immediate health risk [1,2,3,4,5,6]. It is a difficult pathogen to treat because of its strong innate and acquired resistance to known antifungal drugs [3]. Moreover, identifying *C. auris* using traditional microbiological methods is challenging [7]. As a result, the incidence and prevalence of *C. auris* have been increasing among immunocompromised patients and those under long-term hospitalization [1,3,8,9,10]. Owing to its high mortality rate, tendency to cause nosocomial invasive infections, and multidrug-resistant nature, *C. auris* poses a serious global public health concern [11,12,13,14]. *C. auris* has been reported to have a high rate of intrinsic resistance to antifungal treatments such as amphotericin B and fluconazole, but few countries have reported acquired echinocandin resistance [15,16]. In order to prevent a future epidemic, which is probable given the current status of *C. auris*, finding and identifying effective pharmaceutical components to overcome these drug-resistant pathogens must be prioritized [17]. A new antifungal medicine often takes 10 to 15 years to develop; thus, screening molecule libraries can provide a variety of solutions and expedite the identification of candidate drugs [18,19]. Among more than 4300 approved drugs, Cheng et al. reported six anti-*C. auris* compounds that had possible associations with 13 of them [18]. Their screening revealed the potential of amebicide iodoquinol and leishmanicide miltefosine as repositionable compounds, owing to their inhibition of *C. auris* growth [19].

The formation of biofilms (surface-adherent communities) is a major factor in *C. auris* pathogenesis as biofilms contribute to its drug resistance profile. *C. auris* isolates have been detected in several clinical locations, such as central venous catheters, wounds, and stents [20,21]. Biofilms play a crucial role in promoting the persistence and survival of *C. auris* in healthcare facilities as they allow the microorganism to survive on various surfaces for extended periods of time [22]. These factors are responsible for the emergence of *C. auris* as the first leading fungal pathogen to cause an international outbreak in healthcare institutions. Hence, the Centers for Disease Control and Prevention (CDC) released the “Antibiotic Resistance Threats in the United States” report in 2019, which classified *C. auris* as one of five “Urgent Threats” and advocated for assertive and expeditious measures against it [20]. As such, resistance to the limited number of antifungal treatments that are currently available will create various problems in the foreseeable future. Therefore, it is imperative to conduct thorough research on novel pharmaceuticals for combating infections induced by *C. auris*. A suitable strategic approach to acquiring novel antifungal drugs is to explore drug repurposing or repositioning [23,24,25]. For instance, anthelmintic niclosamide has been reported to have an inhibitory effect on *C. auris* biofilm formation. This drug was identified via a screening approach for inhibitors against *C. albicans* and has since been approved by the U.S. Food and Drug Administration (FDA) [26].

Drug repurposing studies are promising for various diseases [27,28,29,30] and have been conducted to identify several active drugs against *C. auris*, such as iodoquinol [20], Miltefosine [20,31], Robenidine [32], Salicylanilide oxyclozanide [33], Pyrvinium pamoate [34], Broxyquinoline [18], Tamoxifen citrate [34], Alexidine dihydrochloride [18,35], AC- 93253 iodide [18], Chloroxine [18], Clioquinol [18], Sertraline [36], Rolipram [34], Trifluoperazine [34], dihydrochloride [34], Thiethylperazine dimalate [34], ebselen [19,34], Disulfiram [37], (−)- MK 801 hydrogen maleate [34], Suloctidil [34], Ciclopirox ethanolamine [34], and Guanadrel sulfate [34]. Nanomaterial-based studies and drug repurposing approaches have led to the proposal of several alternative drugs with *C. aruis* growth inhibition activity. However, an effective treatment is still needed [30,38]. These compounds exhibit frequent activity against new targets that are currently unexplored in antifungal therapies and, consequently, in yeast adaptability. Thus, it is vital to improve infection control procedures by producing effective state-of-the-art disinfectants for managing *C. auris* and opportunistic infection [39]. The identification and development of novel and unique antifungal compounds are of paramount importance in the effective management of *C. auris* infections. While the results obtained using in silico screening approaches are not as precise as those collected from in vivo studies, the cost reduction justifies the efforts. Analyzing 1600 FDA-approved drugs through high-throughput virtual screening and experimental drug repurposing takes much less time compared with conducting in vivo studies for a similar number of drugs. Employing drug repurposing, a potential drug (dutasteride) was identified to inhibit 1,3-beta-glucanosyltransferase, a major cell-wall-associated protein in fungal organisms, including *C. auris* and *Candida albicans*, by retrieving a library of 1615 FDA-approved drugs from the ZINC database. The main objective of this research was to identify novel drugs against *C. auris* and *C. albicans*. This was achieved by performing a virtual screening of 1,3-beta-glucanosyltransferase inhibitors [40,41] to shortlist candidate drugs from the FDA-approved list based on docking scores. This was followed by analyzing the stability of proteins and protein–ligand complexes using molecular dynamics simulations. Then, the uppermost drug with the lowermost binding energy based on drug docking with 1,3-beta-glucanosyltransferase was further explored through an in vitro evaluation of its inhibitory activity against native isolates of *C. auris* and *C. albicans*. Its inhibitory effect was also evaluated by assessing fungal morphology under a scanning electron microscope after treatment with the candidate drug.

## 2. Materials and Methods

### 2.1. FDA-Approved Drugs and Virtual Screening 

The ZINC 20 database [42] [Irwin and Shoichet Laboratories, University of California, USA] is a curated database of 230 million compounds, including FDA-approved drugs, for virtual screening. In addition, ZINC contains over 750 million purchasable compounds that can be searched for analogs in less than a minute. All of the FDA-approved drugs from the ZINC database [42] were obtained and underwent virtual screening to identify potential inhibitors of 1,3-beta-glucanosyltransferase (UniProt: A0A2H1A5Q4), which is one of the surface proteins of *C. auris*. The structure of 1,3-beta-glucanosyltransferase was constructed in PDB format as described earlier using SWISS-MODEL and PYMOL and validated through PROCHECK [43,44]. The FDA-approved drug molecules from the ZINC database and the PDB of 1,3-beta-glucanosyltransferase were imported to the PyRx workspace. PyRx 0.9.9 is a software program used for Computational Drug Discovery through Virtual Screening that enables the screening of compounds from libraries against potential drug targets. Virtual screening was performed individually for each FDA-approved drug molecule against 1,3-beta-glucanosyltransferase using the PyRx 0.9.9 virtual screening tool (https://pyrx.sourceforge.io/ accessed on 28 July 2023). “For computer time, this research used the resources of the Supercomputing Laboratory at King Abdullah University of Science & Technology (KAUST) in Thuwal, Saudi Arabia”.

To understand the behavior of the protein–ligand complex’s 1,3-beta-glucanosyltransferase from *C. auris* and dutasteride within a biological system’s aqueous environment, we employed molecular dynamics simulations. We simulated the docked conformation with the highest affinity for the target protein using Desmond software (version 4.1). The simulation used a TIP3P water model with a cubic box and a 10 Å side length. To maintain electrical neutrality, the system’s net charge was neutralized. We performed the simulations in the NPT ensemble, mimicking constant pressure (1 bar) and temperature (300 K) using the Berendsen coupling scheme, and acquired the final poses for the interacting 1,3-beta-glucanosyltransferase–dutasteride complex at the end. We ran a 100 ns production simulation for the protein–ligand complex after a 100 ns equilibration phase. The stability of the complex was confirmed by the root-mean-square deviation (RMSD) data of the 1,3-beta-glucanosyltransferase–dutasteride complex, dutasteride’s fit on 1,3-beta-glucanosyltransferase, and the development of hydrogen bonds, as well as hydrophobic and water bridges, during the interaction in the simulated 100 ns trajectories.

### 2.2. C. auris and C. albicans Strains

Clinical isolates of two organisms were used in this study including (1) *C. auris* (CA1, CA2, CA3, CA6, CA7, and CA8) and (2) *C. albicans* (CAL). *C. auris* isolates were previously collected from healthcare facilities in Saudi Arabia and identified as having multidrug-resistance mutations [38,45]. The *C. auris* and *C. albicans* (CAL) strains were reconfirmed using the 18S *rRNA* Gene and the ITSa and ITSb regions, as described earlier [38]. Multidrug-resistant *C. auris* CA1 and the clinical isolate *C. albicans* CAL were used for initial primary screening of the selected clinically approved drugs. Follow-up experiments were performed on the *C. auris* (CA1, CA2, CA3, CA6, CA7, and CA8) and *C. albicans* (CAL) strains. *C. auris* and *C. albicans* cultures were cultivated for 24 h by introducing cells into 10 mL of Sabouraud Dextrose Broth (SDB) medium in 150 mL flasks. The flasks were then placed in an orbital shaker operating at a speed of 150 to 180 revolutions per minute (rpm) and maintained at a temperature of 30 °C. After 18 h of incubation, 0.5 × 10^5^ cells/mL were used for the planktonic assay and 1 × 10^6^ cells/mL were suspended in RPMI medium for the biofilm assay (pH 6.9).

### 2.3. Primary Screening for Anti-C. auris Activity 

Cell suspensions of *C. auris* and *C. albicans* were prepared and adjusted to a 0.5 McFarland standard to be used for the initial screening via the Kirby–Bauer disk diffusion method. By measuring the zone of inhibition, the antifungal susceptibility of DR1 [zinc000242548690 digoxin] and DR2 [zinc000003932831 dutasteride] to *C. auris* CA1 *and C. albicans* CAL was determined. Digoxin (0.250 mg tablet, Aspen Bad Oldesloe Gmbh, Bad Oldesloe, Germany) and dutasteride (0.5 mg capsules, GlaxoSmithKline, Poznan, Poland) were used in this study. Initially, 250 µg of dutasteride and 500 µg of digoxin were prepared separately in 1 mL of sterile distilled water as working stock solutions and stored at 4 °C. The antifungal susceptibility screening using the Kirby–Bauer disk diffusion method was performed in triplicate, and fluconazole was tested as a positive control for anti-*C. auris* and anti-*C. albicans* activities.

### 2.4. Determination of Anti-Candida Activity

The anti-*C. auris* and anti-*C. albicans* activities of the selected clinically approved drug dutasteride were analyzed in 96-well microtiter plates via the broth microdilution technique using dose–response assays, as described earlier, to inhibit planktonic growth and biofilm formation [19]. The screening was performed in quadruplicate. 

### 2.5. Dose–Response Assays of Dutasteride-Mediated Inhibition of Planktonic Growth 

The antifungal activity of medicines selected as positives through the initial screening was evaluated using dose–response tests, which measured their inhibition of the planktonic growth of five clinical strains of *C. auris* and one isolate of *C. albicans*. The starting concentration of the selected drugs was 32 μg/mL, and 0.0625 μg/mL was the final concentration achieved using sequential 2-fold dilutions performed across the 96-well microtiter plate rows. Additionally, positive (untreated) and negative controls (uninoculated SDB without *Candida*) were included, and all assays were performed in quadruplicate at each dose in the 96-well plate. After 24h in a shaking incubator at 35 °C, the cells in each well were homogenized, and a microtiter plate reader was used to determine the absorbance at 550 nm (OD550). A dose–response curve was constructed by converting the spectrophotometric measurements into normalized responses. The readings obtained from the positive and negative control wells were arbitrarily assigned growth values of 100% and 0%, respectively. The IC_50_, which represents the concentration of dutasteride required to inhibit 50% of planktonic growth, was determined by employing a linear equation (y = mx + n) to analyze the graph. Specifically, the y value corresponding to 50% inhibition was identified as the IC_50_ value using Microsoft Excel Spreadsheet Software (Microsoft Office Professional Plus 2016, Microsoft, Redmond, WA, USA), and the normalized values were fitted to a variable slope.

### 2.6. Assessment of Biofilm Inhibition Using Dutasteride 

The biofilm biomass of the five *C. auris* clinical strains and one *C. albicans* isolate was assessed using the previously described method of crystal violet staining [19]. The dutasteride concentrations used for this assay ranged from 32 to 0.0625 μg/mL. Briefly, following biofilm formation, PBS was used to wash the plates once, and individual wells were fixed with 100 μL of methanol for 20 min. After removing the methanol, the plates were left to air dry. Then, 150 μL of 0.1% (wt/vol) crystal violet was used to stain the adherent biofilms for 10 min. After the removal of the crystal violet stain, the plates were left to dry and then washed (3 times) with 200 μL of distilled water. An inverted microscope (Olympus CKX41, Tokyo, Japan) was used to directly observe the stained samples on the 96-well plate. To estimate biomass, the crystal violet dye was dissolved by adding 100 μL of 33% glacial acetic acid to each well. The resulting solution was moved to a blank microtiter plate in order to measure the optical density at 550 nm (OD_550_). This measurement was used to quantify the degree of biofilm inhibition in comparison to the untreated control samples.

### 2.7. Assessment of Dutasteride-Treated C. auris and C. albicans Morphology Using SEM

The Kirby–Bauer disk diffusion method was utilized to assess the morphology of *C. auris* and *C. albicans* in SEM. After 24h of incubation with dutasteride, cells were collected from the following regions: the edge of the zone of inhibition by dutasteride against *Candida* [T1], the zone of inhibition by dutasteride against *Candida* [T2], and the usual growth region of *Candida* [T3]. The collected cells were transferred to separate tubes containing 500 µL of 3% glutaraldehyde, incubated for 16 h at 4 °C, and washed 3 times with distilled H_2_O for 10 min. After that, 1% osmium tetroxide (OsO4) was used to fix the samples for 16 h at 4 °C; then, the cells were washed with distilled water for 10 min followed by treatment with a series of acetone concentrations (30%, 50%, 70%, and 100%) for 10 min. Subsequently, the samples were placed in a solution of absolute acetone to facilitate the drying process. The sample was dried using a Leica EM CPD300 automated critical point drying apparatus, which utilizes a combination of acetone and carbon dioxide. The samples were sputter-coated with gold (Q150R ES machine), and double-sided carbon tape was used to mount them. *C. auris* and *C. albicans* cells were observed under a scanning electron microscope (Vega 3, TESCAN, Brno, Czech Republic) operated at 20 kV. The digitized photos were subjected to analysis using ImageJ 1.53t software [46].

## 3. Results

### 3.1. Virtual Screening for Anti-C. auris Activity

**Figure 1:**
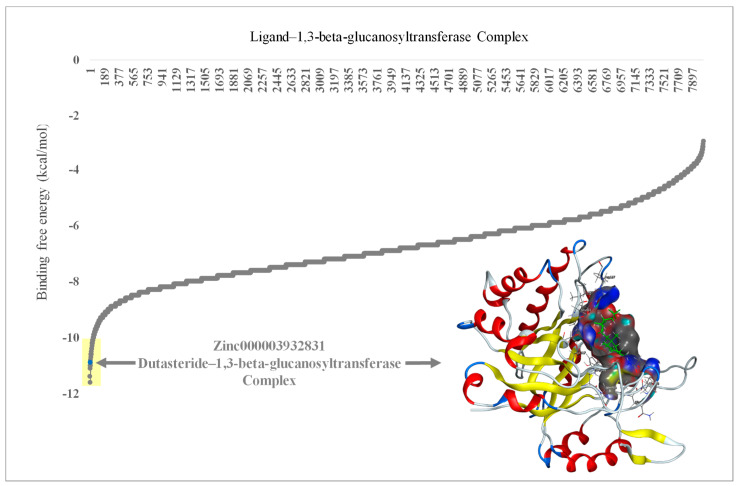
Molecular docking results of 1,3-beta-glucanosyltransferase (UniProt: A0A2H1A5Q4) from *C. auris* and the FDA-approved drug complex, including energy minimization. The arrow indicates the lowest binding affinity of −11 Kcal/mol. Yellow shade indicates the lowest binding affinity of ≤−10 Kcal/mol in ligands zinc000242548690 (digoxin), zinc000003932831 (dutasteride), zinc000052955754 (ergotamine), and zinc000203757351 (paritaprevir). The embedded protein–ligand complex is the 1,3-beta-glucanosyltransferase–dutasteride complex.

**Figure 2:**
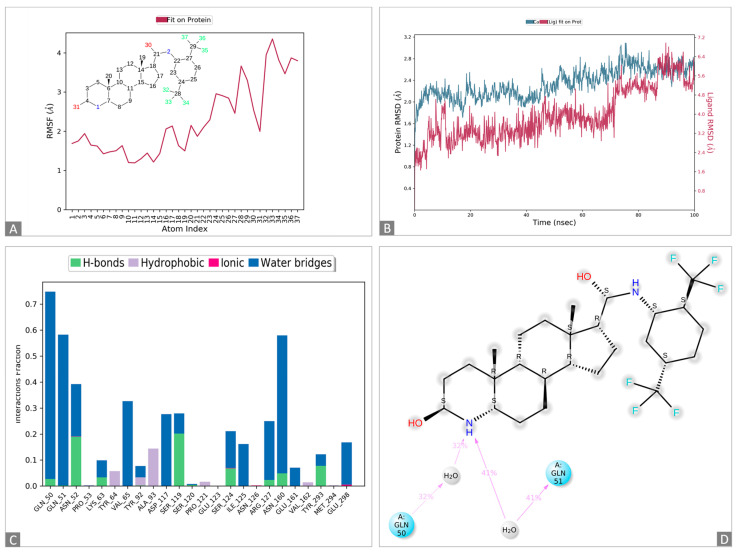
Molecular dynamics simulations of the 1,3-beta-glucanosyltransferase (UniPort: A0A2H1A5Q4)-and-dutasteride complex. (**A**): Ligand dutasteride fluctuations with respect to 1,3-beta-glucanosyltransferase. (**B**): Protein–ligand root-mean-square deviation (RMSD) of 1,3-beta-glucanosyltransferase and dutasteride. (**C**): Types of 1,3-beta-glucanosyltransferase–dutasteride interactions or “contacts” of 1,3-beta-glucanosyltransferase and dutasteride. (**D**): Dutasteride atom interactions with 1,3-beta-glucanosyltransferase and dutasteride.

FDA-approved drugs (*n* = 1600) were obtained from the ZINC database and analyzed through virtual screening to detect inhibitors of 1,3-beta-glucanosyltransferase. The calculated binding affinities of the 1,3-beta-glucanosyltransferase-containing FDA-approved drugs were arranged from lowest to highest to identify the lowermost binding affinity (Figure 1). Ligands such as zinc000242548690 (digoxin), zinc000003932831 (dutasteride), zinc000052955754 (ergotamine), and zinc000203757351 (paritaprevir) were found to have a binding affinity of ≤−10 Kcal/mol. The two ligands [DR1: zinc000242548690 (digoxin) and DR2: zinc000003932831 (dutasteride)] with the lowermost binding affinity were selected for the in vitro evaluation of anti-*C. auris* and anti-*C. albicans* activity. The ligand DR2 (dutasteride) showed anti-*C. auris* and anti-*C. albicans* activity against the native fungal isolates, multidrug-resistant *C. auris* and *C. albicans.* However, DR1 showed no anti-*Candida* activity. Hence, DR2 was further explored by analyzing the stability of the 1,3-beta-glucanosyltransferase–DR2 complex through molecular dynamics (MD) simulations (Figure 2). 

The docked complexes of 1,3-beta-glucanosyltransferase from *C. auris* and dutasteride were analyzed to assess their intermolecular interaction and stability with respect to time via a 100-nanosecond molecular dynamics simulation, and the final poses for the interacting 1,3-beta-glucanosyltransferase–dutasteride complex were acquired at the end (Figure 2 and Figure S1). The MD simulations of 1,3-beta-glucanosyltransferase–dutasteride confirmed the interaction in the docked complex. The root-mean-square deviation (RMSD) data of the 1,3-beta-glucanosyltransferase–dutasteride complex, dutasteride’s fit on 1,3-beta-glucanosyltransferase, and the development of hydrogen bonds, as well as hydrophobic and water bridges, during the interaction in the simulated 100 ns trajectories confirmed the stability of the complex. Metabolites of dutasteride, including 4′-hydroxydutasteride, 6-hydroxydutasteride, and 6,4′-dihydroxydutasteride, as well as a minor metabolite, 15-hydroxydutasteride, were tested for 1,3-beta-glucanosyltransferase–dutasteride metabolite interactions using virtual screening software. The interaction in the docked complex of the drug metabolite and 1,3-beta-glucanosyltransferase confirmed the high binding affinity of dutasteride metabolites, 6,4′-dihydroxydutasteride (binding affinity = −10.1), 6-hydroxydutasteride (binding affinity = −9.6), and 4′-hydroxydutasteride (binding affinity = −9.5) with the metabolites of dutasteride, except the minor metabolite, 15-hydroxydutasteride (binding affinity = −1.7). 

### 3.2. Morphological Changes upon Dutasteride Treatment

**Figure 3:**
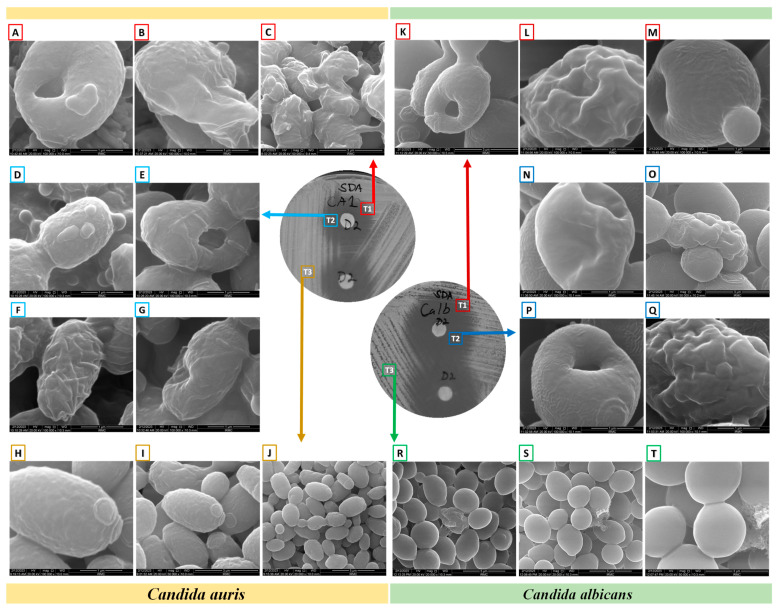
Morphology of *C. albicans* and *C. auris* under a scanning electron microscope after determining their antifungal susceptibilities to dutasteride. (**A**–**C**,**K**–**M**): Morphologies of *Candida* from the edge of the zone of inhibition (ZOI) [T1]. (**A**–**C**): Changes in the morphologies of *C. auris* from the edge of the ZOI [T1] after treatment with dutasteride. (**D**–**G**,**N**–**Q**): *Candida* cells from the ZOI [T2]. (**D**–**G**): Changes in the morphologies of *C. auris* from the ZOI [T2] after treatment using dutasteride. (**H**–**J**): Structure of *C. auris* from the area of standard growth [T3]. (**K**–**M**): Changes in the morphologies of *C. albicans* from the edge of the ZOI [T1] after treatment using dutasteride. (**N**–**Q**): Changes in the morphologies of *C. albicans* from the ZOI [T2] after treatment using dutasteride. (**R**–**T**): Structure of *C. albicans* from the area of standard growth [T3].

**Figure 4:**
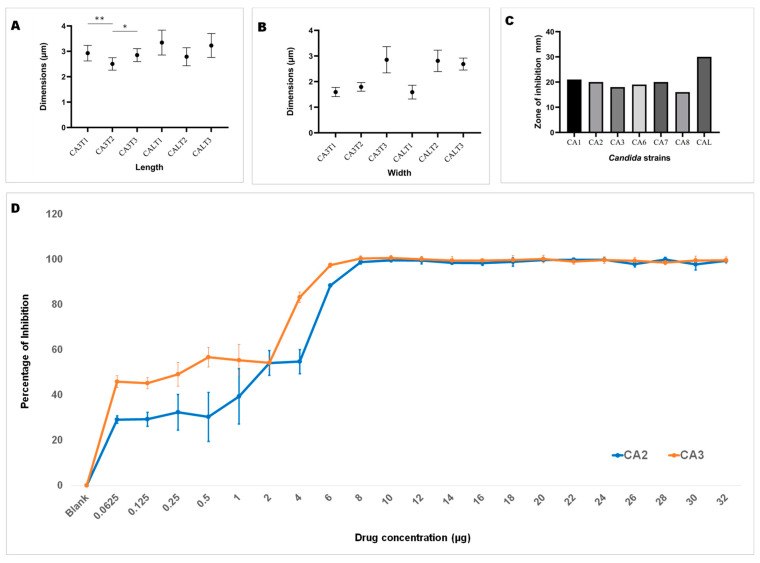
Length and width of *C. auris* and *C. albicans*. (**A**): Length of *C. auris* (CA3) and *C. albicans* (CAL) cells after treatment using dutasteride (CA3T1, CA3T2, CALT1, and CALT2) and cells without treatment, i.e., the control (CA3T3 and CALT3). * Significant at a *p*-value of <0.05; ** significant at a *p*-value of <0.01. (**B**): Width of *C. auris* (CA3) and *C. albicans* (CAL) cells after treatment using dutasteride (CA3T1, CA3T2, CALT1, and CALT2) and *Candida* cells without treatment, i.e., the control (CA3T3 and CALT3). (**C**): Observation of the zone of inhibition resulting from dutasteride treatment against *C. auris* and *C. albicans*. (**D**): Inhibition of biofilm formation of *C. auris* CA2 and *C. auris* CA3 clinical isolates by dutasteride. Graphs illustrate the percentage of biofilm formation inhibition by dutasteride against *C. auris* CA2 and *C. auris* CA3. All assays of biofilm formation inhibition were executed in quadruplicate in a 96-well plate. Error bars in the graphs designate standard errors.

Upon ensuring the stability of the complex (1,3-beta-glucanosyltransferase–dutasteride), the morphological impacts of dutasteride on *C. auris* and *C. albicans* cells were analyzed using SEM. Antifungal susceptibility was assessed based on morphological changes in the structure (Figure 3) and dimensions (length, width) of *Candida* cells (Figure 4) after dutasteride treatment. One-way ANOVA in the horizontal dimension of *C. auris* cells (CA3T1: length 2.9294 ± 0.3072 µm; width = 1.5922 ± 0.1787 µm; CA3T2: length 2.5045 ± 0.2447 µm; width 1.7929 ± 0.1675 µm; CA3T3: length 2.8502 ± 0.2540 µm; width 2.8527 ± 0.5110 µm) revealed significant (F statistic = 6.2990; *p*-value = 0.0057) differences in length. One-way ANOVA in the horizontal dimension of *C. albicans* cells (CALT1: length 3.3452 ± 0.4906 µm; width 1.5892 ± 0.2680 µm; CALT2: length 2.7874 ± 0.3549 µm; width 2.8111 ± 0.4180 µm; CALT3: length 3.2296 ± 0.4716 µm; width 2.6848 ± 0.2331 µm) also showed significant (F statistic = 3.9722; *p*-value = 0.0307) differences in length (Figure 4). However, the SEM analysis of width after dutasteride treatment revealed insignificant changes in the *Candida* cells. The dutasteride treatment resulted in clear morphological changes indicated by cell membrane disruptions and lysed *Candida* cells. Furthermore, the SEM image confirmed the smooth phenotype of the healthy *C. auris* and *C. albicans* cells and the crushed phenotype of *C. auris* and *C. albicans* after dutasteride treatment. The positive control fluconazole was tested for anti-*C*. *auris* and anti-*C*. *albicans* activities, and both *C*. *auris* and *C*. *albicans* showed resistance to it.

### 3.3. Dose–Response Assays of Dutasteride’s Inhibition of Planktonic Growth

Using the results of dutasteride dose–response curves (32 to 0.0625 µg/mL) to inhibit the planktonic growth of *C. auris* CA1, CA2, CA3, CA5, CA6, CA7, and CA8 and *C. albicans* CAL, we determined the 50% inhibitory concentration (IC_50_) (Table 1). The results revealed an IC_50_ of 8.6833 ± 1.832 µg/mL [CA1: 6.0097 µg; CA2: 6.5264 µg/mL; CA3: 10.3217 µg/mL; CA5: 9.5679 µg/mL; CA6: 10.1056 µg/mL; CA7: 10.2367 µg/mL; and CA8: 8.0151 µg/mL] for *C. auris* and an IC_50 of_ 10.8116 µg/mL for *C. albicans*.

### 3.4. Assessment of Biofilm Inhibition Using Dutasteride 

Dutasteride was effective at inhibiting biofilm formation by *C. auris* and *C. albicans* (Figure 4D). Dutasteride revealed narrow variations in its inhibition of biofilm formation by the seven *C. auris* clinical isolates and one *C. albicans* clinical isolate. The formation of biofilm by *C. auris* CA8, CA7, and CA3 was efficiently inhibited by dutasteride compared with the inhibition of biofilm formation by *C. albicans* CAL (Figure 4D). The inhibiting ability of dutasteride against the formation of biofilm by *C. auris* CA1, CA2, CA6, and CA5 was reduced at lower concentrations of dutasteride. More than 98% of biofilm formation by the six tested clinical isolates, including *C. auris* CA3, CA5, CA6, CA7, and CA8 and *C. albicans* CAL, was inhibited by dutasteride at the highest concentration tested (Figure 4D). We determined the IC_50_ at which 50% biofilm formation was inhibited by dutasteride. The average biofilm IC_50_ for the tested clinical isolates of *C. auris* ranged from 4.840 to 11.632 µg/mL, with an average of 7.282 ± 2.799 µg/mL. The biofilm IC_50_ for the clinical isolate of *C. albicans* was 6.310 µg/mL (Table 1). The 1,3-beta-glucanosyltransferase–dutasteride complex showed lower binding free energy in the virtual screening results, which was confirmed by the MD simulations. Dutasteride’s activity in inhibiting the biofilm formation and planktonic growth of *C. auris* confirmed these predictions, indirectly indicating stability and a stronger interaction.

## 4. Discussion 

Drug repurposing is a suitable method for finding new uses for drugs that have been previously researched and registered [47]. It can help avoid significant expenses related to the research and development of new drugs, and it also uses de-risked compounds to reduce attrition rates. As a result, this method has been used to repurpose medications against *C. auris*. The selection of appropriate target proteins is an important factor in virtual screening. This study considered Gibbs free energy and binding free energy by assessing negative values, which indicated whether a drug is predicted to bind to 1,3-beta–glucanosyltransferase in *C. auris*. We selected 1,3-beta-glucanosyltransferase, a major cell-wall-associated protein in genera like *Candida* [40,41]. The target 1,3-beta-glucanosyltransferase in *C. auris* was successfully inhibited by various FDA-approved drugs during virtual screening. 

Through virtual screening, we found that a lower binding free energy indicates more stability and a stronger interaction, and we confirmed its potential association with higher drug efficacy using MD simulations and its activity in biofilm formation and planktonic growth. Specific interactions, including hydrogen bonding and other interactions between 1,3-beta-glucanosyltransferase and dutasteride, revealed improved binding. The conformational changes in MD simulations revealed how 1,3-beta-glucanosyltransferase might shift upon dutasteride binding, which signifies that dutasteride might work effectively when 1,3-beta-glucanosyltransferase adopts a specific conformation. Hydrogen bonding between the first amino group of dutasteride and the carbonyl group of glutamine (the 50th or 51st amino acid) can be crucial for understanding how dutasteride binds to 1,3-beta-glucanosyltransferase and designing new drugs. The observations made through MD simulations in this study may be useful for identifying new drug targets using 1,3-beta–glucanosyltransferase in *C. auris* and can predict off-target effects in the future by virtually screening existing drugs followed by wet-lab studies against various protein targets.

The four top hits [zinc000242548690 (digoxin C_41_H_64_O_14_), zinc000003932831 (dutasteride C_27_H_30_F_6_N_2_O_2_), zinc000052955754 (Ergotamine C_33_H_35_N_5_O_5_), and zinc000203757351 (Paritaprevir C_40_H_43_N_7_O_7_S)] are unrelated drugs, which were initially selected based on virtual screening for the inhibitors of 1,3-beta-glucanosyltransferase from *C. auris*. Digoxin (C_41_H_64_O_14_) is a cardiac glycoside used to treat moderate heart failure. Dutasteride is an antiandrogenic compound that inhibits 5-alpha reductase and is used to treat benign prostatic hyperplasia in adult males. Cluster headaches and migraines are treated with ergotamine, which is an alpha-1-selective adrenergic agonist vasoconstrictor. A direct-acting antiviral drug called paritaprevir is combined with other antiviral drugs to treat infections caused by the hepatitis C virus [48]. In the present study, we used clinical isolates of *C. auris* from Saudi Arabia, previously confirmed by our team as having various drug-resistance mutations, and all the strains were reconfirmed using molecular techniques [38,45]. We used the multidrug-resistant clinical isolate *C. auris* CA1 for the initial primary screening in parallel with the clinical isolate *C. albicans* CAL for inhibiting the activity of the top-rated (highlighted in yellow in Figure 1) clinically approved drugs from the virtual screening. As the major aim of this research was to identify FDA-approved drugs with anti-*C. auris* activity for use in drug repurposing, we focused on the top two drugs, i.e., zinc000242548690 (digoxin) and zinc000003932831 (dutasteride), from the highest predications. Furthermore, no antifungal drugs from the tested groups were in the top-rated category, with binding affinities of ≤−10 Kcal/mol. Here, the ability of *C. auris* to live on hospital surfaces and places other than the human body should be considered, and appropriate environmental decontamination and surface disinfection methods should be developed for hospitals and research labs to prevent the emergence of nosocomial pathogens like *C. auris* [19,49,50]. Even though zinc000242548690 digoxin (DR1) was rated highest in the prediction of binding affinity, it showed no anti-*Candida* activity, which was in line with earlier observations of *C. albicans* and *Saccharomyces cerevisiae* [51]. One hypothesis is that the cell wall or plasma membrane of *C. albicans* and *C. auris* prevented digoxin from entering the cytoplasm [51,52]. Digoxin, a cardiac glycoside, is a medication for heart conditions such as congestive heart failure and certain cardiac arrhythmias and has a narrow therapeutic window [53]. Our study once again confirms this, as despite a positive virtual screening result for binding, no wet-lab studies confirmed digoxin activity against *C. auris* cells. Digoxin is a toxic substance with a well-known cardiotoxic effect. Despite digoxin’s long-standing approval, it is important to acknowledge its potent side effects. If overdosed, the consequences can be severe [53]. This study confirmed a lack of anti-*C. auris* activity in fluconazole, as reported earlier by various studies [19]. 

zinc000003932831 dutasteride (DR2) was rated the second highest in the prediction of binding affinity, with anti-*Candida* activity confirmed in the primary screening. To our knowledge, this is the first report providing evidence for the anti-*C. auris* activity of dutasteride. A randomized, double-blind, placebo-controlled interventional trial observed reduced viral shedding in males with mild COVID-19 treated with dutasteride and other drugs [54]. More confirmative studies are needed to ensure that dutasteride will eventually be a more effective anti-*C. auris* treatment than the existing drugs or will have more promising antifungal potential. The primary screening of dutasteride on *C. auris* was extended to confirm its anti-*C. auris* activity through dose–response assays, over a range of concentrations (32 to 0.065 µg/mL) of dutasteride, against different clinical isolates of *C. auris*, and we calculated the IC_50_ against various clinical isolates of *C. auris* and *C. albicans*. Dutasteride at an 8.949 µg/mL concentration was able to inhibit 50% of the growth of clinical isolates of *C. auris.* The IC_50_ was used to identify the effective quantifying mechanism and revealed the half-maximum inhibitory concentration of dutasteride in inhibiting the growth of *C. auris*. It can also be used to determine appropriate dutasteride dosages for further testing and development studies. Regarding the inhibition of the planktonic growth of C. auris, the IC_50_ refers to the concentration of dutasteride needed to inhibit the growth of free-floating *C. auris* cells in broth culture. Biofilms are essential structural characteristics of *C. auris* attached to surfaces; the IC_50_ signifies the concentration of dutasteride required to disrupt an existing biofilm of *C. auris* and the formation of new biofilm by *C. auris*. The IC_50_ values observed for planktonic growth do not have a direct association with biofilm formation; however, they have a considerable influence on our understanding of the potential effectiveness of dutasteride.

The results of the electron microscopic studies clearly indicate that dutasteride is fungicidal. In order to identify potential drugs, screening libraries can provide a number of solutions. Through the Prestwick Chemical Library, a repurposing library, ebselen, which is an antioxidant, anti-inflammatory, and cytoprotective drug, was identified as a repositionable molecule and inhibited the growth of *C. auris* as well as biofilm formation [19]. A systemic review revealed studies about 12 repositionable drugs/compounds that inhibit the formation of *C. auris* biofilms, which included antiparasitic drugs (iodoquinol, Miltefosine, Niclosamide, Tri-Chloro-Salicyanilide), anti-inflammatory drugs (ebselen, AM-24 (2,4,6-triiodophenol)), anticancer drugs (Alexidine dihydrochloride), antidepressants (Sertraline), psychiatric agents (Disulfiram), a muscarinic receptor agonist (Tazomeline), a Farnesyl transferase inhibitor (Lonafarnib), and others (Provecta—rose bengal disodium) [18,30,31,32,33,35,36,37]. In addition, they revealed 22 repositionable drugs/compounds that inhibit planktonic growth in *C. auris*, which included antiparasitic drugs (iodoquinol, Miltefosine, Robenidine, Salicylanilide oxyclozanide, Pyrvinium pamoate, Broxyquinoline, Diiodohydroxyquinoline), anticancer drugs (Tamoxifen citrate, Alexidine dihydrochloride, AC-93253 iodide), antiseptics (Chloroxine, Clioquinol), antiemetics (Trifluoperazine: dihydrochloride, Thiethylperazine dimalate), and antidepressants (Sertraline, Rolipram), as well as antiviral drugs (Ribavirin), anti-inflammatory agents (ebselen), antifungal/antibacterial agents (Ciclopirox ethanolamine), anticonvulsants ((−)-MK 801 hydrogen maleate), antihypertensives (Guanadrel sulfate), antiplatelets (Suloctidil), and psychiatric agents (Disulfiram) [18,30,31,32,33,35,36,37]. A large screening study demonstrated 27 drugs that inhibited the growth of three different strains of *C. auris* with different geographical origins. These included antibacterial (Alexidine dihydrochloride, Benzethonium chloride, Chloroxine, Dequalinium dichloride, Methyl benzethonium chloride), antimalarial (Artemisinin), antibacterial/antifungal (Ciclopirox ethanolamine), antiamebic/antibacterial (Clioquinol), antipruritic (Dimethisoquin hydrochloride), local anesthetic (Dyclonine hydrochloride), anti-inflammatory (ebselen), anti-fatigue (Fipexide hydrochloride), antihypertensive (Guanadrel sulfate), antiseptic (Hexachlorophene, Thonzonium bromide), antipsychotic (Methiothepin maleate, Zotepine), anticonvulsant (MK 801 hydrogen maleate), anthelmintic (Pyrvinium pamoate), antiamebic/antipsychotic (Prochlorperazine dimaleate), antineoplastic (Tamoxifen citrate), antiemetic (Thiethylperazine dimalate, Trifluoperazine dihydrochloride), antidepressant (Rolipram, Sertraline), and antiplatelet (Suloctidil) drugs [34]. In addition to these drugs, the present study considers dutasteride an effective anti-*Candida* agent with promising inhibition of the planktonic growth of *C. auris* and *C. albicans* while also inhibiting biofilm formation by both *Candida* species. A smaller amount of dutasteride is needed to inhibit 50% of the biofilm formation in *C. auris* and *C. albicans* isolates, compared with 50% of planktonic growth for the strains *C. auris* CA3, CA5, CA6, CA7, and CA8 and *C. albicans*. However, it is different for the strains *C. auris* CA1 and CA2. The biofilm-inhibiting activity of dutasteride indicates the competency of the drug to fight the high-level resistance characteristics of *C. auris* and *C. albicans* [19,55,56,57,58]. On all of the tested clinical isolates of *C. auris* and *C. albicans*, in addition to biofilm and planktonic growth inhibition by dutasteride, we demonstrated dutasteride-induced morphological changes like cell disruption, a crushed phenotype structure, and a significant reduction in length. 

In 2002, clinical research demonstrated the effectiveness of dutasteride at a dosage of 0.5 mg per day. The research findings indicated that dutasteride medication resulted in a smaller increase in adverse sexual events compared with a placebo. Nevertheless, its extended usage for a period exceeding 4 years did not result in an escalation of the adverse effects that were identified throughout the initial 2-year investigation. Hence, dutasteride not only enhances urinary symptoms and flow rate but is also linked to noteworthy enhancements in benign prostatic hyperplasia Impact Index scores, indicating benefits to quality of life for men with benign prostatic hyperplasia. During a 2-year period, a minimum of 1% of patients who received either dutasteride or a placebo experienced side effects, including impotence, ejaculation problems, decreased libido, and gynecomastia. Nevertheless, when dutasteride is used daily for a maximum of 2 years, its tolerability profile is similar to that of a placebo, with the exception of slightly higher occurrences of gynecomastia, impotence, and lower libido. Remarkably, the occurrence of the majority of drug-induced sexual side effects declined in individuals who were administered dutasteride consistently over the course of 48 months. Overall, long-term treatment with dutasteride does not result in an increased occurrence of negative outcomes, and patients generally adhere well to the treatment [59,60]. It is important to take these data into account when evaluating the future development of high dosages of dutasteride to treat individuals with *C. auris* infection. The liver extensively metabolizes dutasteride, primarily excreting it in the feces, and trace amounts are excreted in urine [61]. Researchers have identified *C. auris* in urinary tract infections worldwide. Therefore, we can consider dutasteride as a possible substitute if *C. auris* infection persists or recurs; however, the need for higher concentrations should not be ignored [62]. These observations confirm the need for further animal model studies and clinical trials to investigate dutasteride as a potential anti-*Candida* drug to treat *C. auris* and *C. albicans* infections.

## 5. Conclusions 

The present study used high-throughput virtual screening of drug repurposing to significantly reduce costs and save time in identifying novel and effective anti-*Candida* drugs against the nosocomial pathogen *C. auris*, which exhibits multidrug resistance and is an emerging pathogen of increasing concern. The drug repurposing of dutasteride, which has potential antifungal efficiency (specifically with anti-*C. auris* and anti-*C. albicans* activity), could enable the rapid recovery of patients infected with multidrug-resistant *C. auris*. Furthermore, detailed investigations on dutasteride’s non-antifungal off-target effects using animal model studies are needed before it can be considered a repositionable clinical agent to reduce nosocomial infections and alleviate difficult-to-treat candidiasis and molds.

## Figures and Tables

**Table 1:** The planktonic and biofilm inhibition results.

Strain	Inhibition of Planktonic Growth (IC_50_ µg/mL)	Inhibition of Biofilm Formation (IC_50_ µg/mL)
*C. auris* CA1	6.01	11.63
*C. auris* CA2	6.53	10.34
*C. auris* CA3	10.32	4.90
*C. auris* CA5	9.57	6.19
*C. auris* CA6	10.11	8.08
*C. auris* CA7	10.24	4.84
*C. auris* CA8	8.02	4.99
*C. albicans* CAL	10.81	6.31

## Data Availability

Data are contained within the article and Supplementary Materials.

## Supplementary Materials

The following supporting information can be downloaded at https://www.mdpi.com/article/10.3390/pharmaceutics16060810/s1, Figure S1. Molecular dynamics simulations of the 1,3-beta-glucanosyltransferase (UniPort: A0A2H1A5Q4)-and-dutasteride complex. A: Structure (2D) of dutasteride (DrugBank: DB01126); B: root-mean-square fluctuation (RMSF) in protein (P-RMSF). D: Ligand dutasteride fluctuations with respect to 1,3-beta-glucanosyltransferase. C: Protein secondary structure element (P-SSE) timeline. D: 1,3-beta-glucanosyltransferase–dutasteride contact timeline. E: A dutasteride torsion plot of the conformational changes in every rotatable bond in dutasteride in the simulation (0.00–100.00 nanoseconds) trajectory. The histogram displays the conformational strain dutasteride undergoes to maintain a 1,3-beta-glucanosyltransferase-bound conformation. The colors in the inserted structure of dutasteride correspond to the rotatable bonds of dutasteride as well as the dial plot and bar plots.
