# Supplementary material for: The Potential of Dutasteride for Treating Multidrug-Resistant Candida auris Infection
Source: Pharmaceutics. 2024 Jun 14;16(6):810. doi: 10.3390/pharmaceutics16060810 (PMC11207579; doi:10.3390/pharmaceutics16060810)
Supplement: Supplementary file 1 [file pharmaceutics-16-00810-s001.zip › pharmaceutics-3004033-supplementary.pdf]

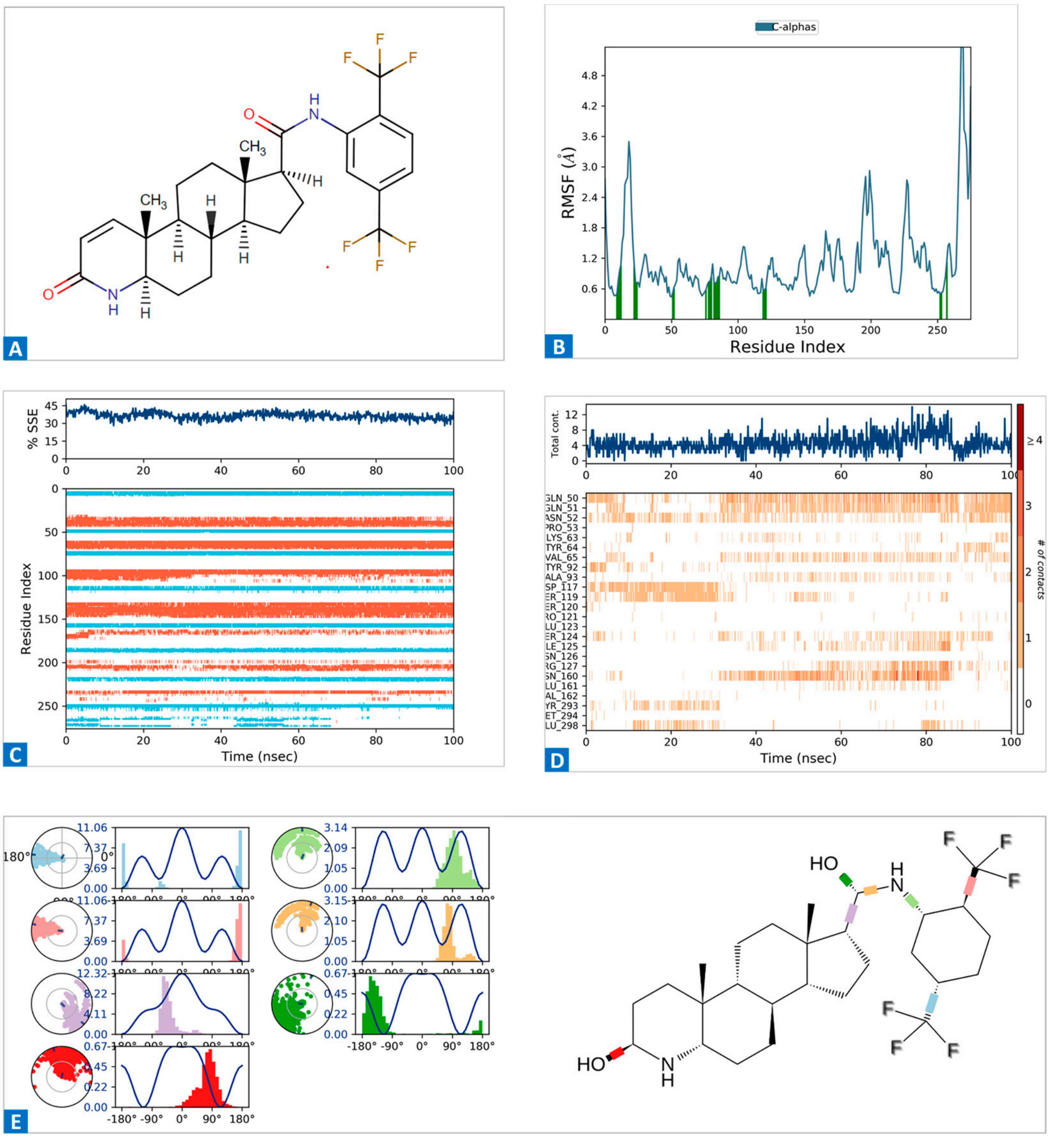

Figure S1. Molecular dynamics simulations of the 1,3-beta-glucanosyltransferase (UniPort: A0A2H1A5Q4)-and-dutasteride complex. A: Structure (2D) of dutasteride (DrugBank: DB01126); B: root-mean-square fluctuation (RMSF) of protein (P-RMSF). D: Ligand dutasteride fluctuations with respect to 1,3-beta-glucanosyltransferase. C: Protein secondary structure element (P-SSE) timeline. D: 1,3-beta-glucanosyltransferase–dutasteride contact timeline. E: A dutasteride torsion plot of the conformational changes of every rotatable bond in dutasteride in the simulation (0.00–100.00 nanoseconds) trajectory. Histogram displays the conformational strain the dutasteride undergoes to maintain a 1,3-beta-glucanosyltransferase-bound conformation. The colors in the inserted structure of dutasteride correspond to the rotatable bonds of dutasteride as well as the dial plot and bar plots.
